# Exploring Solar Cells Based on Lead- and Iodide-Deficient Halide Perovskite (d-HP) Thin Films

**DOI:** 10.3390/nano13071245

**Published:** 2023-03-31

**Authors:** Liam Gollino, Nicolas Mercier, Thierry Pauporté

**Affiliations:** 1 Institut de Recherche de Chimie-Paris (IRCP), UMR8247, CNRS, Chimie-ParisTech, PSL Université, 11 rue Pierre et Marie Curie, CEDEX 5, 75231 Paris, France; 2 MOLTECH-Anjou, UMR 6200, University of Angers, 2 boulevard de Lavoisier, 49045 Angers, France

**Keywords:** perovskite solar cells, lead- and iodide-deficient perovskite, hydroxyethylammonium, thioethylammonium, stability

## Abstract

Perovskite solar cells have become more and more attractive and competitive. However, their toxicity induced by the presence of lead and their rather low stability hinders their potential and future commercialization. Reducing lead content while improving stability then appears as a major axis of development. In the last years, we have reported a new family of perovskite presenting PbI^+^ unit vacancies inside the lattice caused by the insertion of big organic cations that do not respect the Goldschmidt tolerance factor: hydroxyethylammonium HO-(CH_2_)_2_-NH_3_^+^ (HEA^+^) and thioethylammonium HS-(CH_2_)_2_-NH_3_^+^ (TEA^+^). These perovskites, named d-HPs for lead and halide-deficient perovskites, present a 3D perovskite corner-shared Pb_1−x_I_3−x_ network that can be assimilated to a lead-iodide-deficient MAPbI_3_ or FAPbI_3_ network. Here, we propose the chemical engineering of both systems for solar cell optimization. For d-MAPbI_3_-HEA, the power conversion efficiency (PCE) reached 11.47% while displaying enhanced stability and reduced lead content of 13% compared to MAPbI_3_. On the other hand, d-FAPbI_3_-TEA delivered a PCE of 8.33% with astounding perovskite film stability compared to classic α-FAPI. The presence of TEA^+^ within the lattice impedes α-FAPI degradation into yellow δ-FAPbI_3_ by direct degradation into inactive Pb(OH)I, thus dramatically slowing the aging of d-FAPbI_3_-TEA perovskite.

## 1. Introduction

Hybrid halide perovskites (PVKs) are compounds whose general formula is ABX_3_, where A is a monovalent organic or inorganic cation, mainly Cs^+^, CH_3_NH_3_^+^ (methylammonium, noted MA^+^) or HC(NH_2_)_2_^+^ (formamidinium, noted FA^+^), B is an octahedrally coordinated divalent metal ion (classically Pb^2+^ or Sn^2+^), and X is a halogen (generally Cl^−^, Br^−^ or I^−^) [1,2]. These compounds crystallize in a cubic or tetragonal structure at room temperature [3]. PVKs have attracted great attention from the scientific community worldwide for a large range of optoelectronic applications such as lasers, [4] LEDs, [5] photodetectors, [6,7] scintillation, [8] photocatalysis, [9,10,11] and photovoltaic perovskite solar cells (PSCs) [12,13,14,15,16,17,18,19,20,21].

Since their appearance in 2009, [12] PSCs have emerged as very promising materials for the future of photovoltaic devices [13,14,15,16]. Henceforth, due to their optoelectronic properties such as high absorption coefficients [17], high charge carrier mobility [18], long carrier diffusion lengths [19], low recombination loss [20] and tunable bandgap [21], PSCs have been widely investigated and their PCE have increased apace, to reach a certified record efficiency of 25.7% in 2022 [22,23]. One of the most studied perovskites is methylammonium lead iodide, MAPbI_3_ (noted MAPI) [24,25,26,27,28,29,30,31]. However, the toxicity of halide perovskites induced by the presence of lead [32,33,34,35,36] and their rather low stability [37] hinder their potential and future commercialization. With the aim of solving the first problem, many studies have been implemented to partially or fully substitute lead with other less/non-toxic elements such as Sn, Sr, Cu, Ag, or Ge to transform the reference MAPbI_3_ perovskite into MAPb_1−x_A_x_X_3_ [38,39,40,41,42,43]. Nevertheless, except for Sn substitution, which can reach a high percentage level (up to 50% or more) and still reach high efficiency [44,45], other elements can only be added in very small amounts (less than 5%), which is not sufficient. Regarding the stability issue, studies on encapsulation [46], 2D and quasi-2D perovskites [47], multiplication integration [48,49,50], and barrier layers [51] have been conducted. In particular, recently, many studies have been published about MA-free perovskite that delivers improved efficiencies and stability [52,53]. In the last years, a new family of perovskite presenting PbI^+^ units vacancies inside the perovskite lattice, named d-HPs for “Lead- and Iodide—Deficient Halide Perovskites,” has been discovered that can be considered as a bridge between 3D and 2D perovskites [54,55]. Their general formulation is (A’)_3.48x_(A)_1–2.48x_[Pb_1−x_I_3−x_], where A is methylammonium or formamidinium (FA^+^). Reported A’ are two large organic cations, hydroxyethylammonium HO-(CH_2_)_2_-NH_3_^+^ (HEA^+^) [54] and thioethylammonium HS-(CH_2_)_2_-NH_3_^+^ (TEA^+^) [55], that do not respect the Goldschmidt tolerance factor [56]. They exhibit a 3D perovskite corner-shared Pb_1−x_I_3−x_ network that can be assimilated to a lead-iodide-deficient MAPbI_3_ or FAPbI_3_ (FAPI) network. By inserting HEA^+^ or TEA^+^, it has been possible to develop d-MAPI-HEA and d-FAPI-TEA perovskites, respectively. Each of these systems has undergone few optimizations as thin films yet, but the crucial point is that an improvement in the stability for both systems was observed. Similar structures have been reported based on the use of other large cations such as ethylenediammonium [57,58,59,60,61,62,63], propylenediammonium [64], and trimethylenediammonium [64] by Kanatzidis et al. They have called this family “Hollow” perovskites.

In this article, we explore the employment of d-MAPI-HEA and d-FAPI-TEA thin films for solar cell application, and we compare them to their MAPI and FAPI reference counterparts. Perovskite thin films have been fully characterized by X-ray diffraction (XRD), UV-Visible absorption, steady-state photoluminescence (PL), and Scanning Electron Microscopy (SEM). The stability of thin films and complete PSCs under ambient conditions have also been assessed to finalize the study. Important improvements have been reached for each system. d-MAPI-HEA PSCs deliver an optimized PCE of 11.47%, with improved stability of perovskite films. In the meantime, d-FAPI-TEA PSCs reach a final PCE of 8.33% with astonishingly good stability under ambient conditions compared to α-FAPI PSCs.

## 2. Results and Discussion

**Figure 1:**
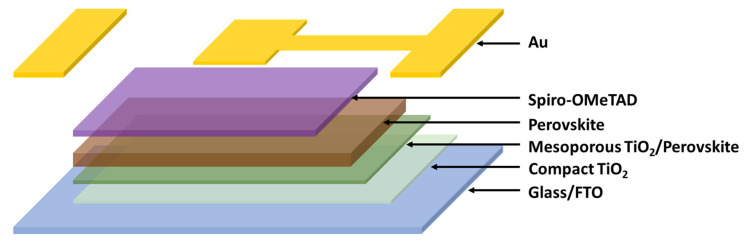
Exploded schematic view of the perovskite solar cell structure.

All the fabricated cells had a direct mesoporous structure, consisting of FTO-coated glass as the substrate, a combination of compact and mesoporous TiO_2_ layers as the electron transporting layer (ETL), perovskite as the absorber, spiro-OMeTAD as the hole transporting layer (HTL) and gold as the counter-electrode (Figure 1). We have tested a large number of parameters for each system and finally developed an optimized recipe for each one. This article is divided into two parts. The first part is dedicated to the d-MAPI-HEA system for which we selected an x = 0.13 value, while the second part is dedicated to the d-FAPI-TEA one with x = 0.04. Such x values have been retained regarding our previous reports [54,55] and preliminary studies in order to obtain a compromise between lead and iodide reduction, efficiency, and stability while retaining the d-HP structure. For both systems, we report the parameters that influence their PCE the most before fully characterizing the final systems.

### 2.1. d-MAPI-HEA Based Solar Cells

For d-MAPI-HEA (x = 0.13), we first focused on the impact of DMF/DMSO mixtures as a solvent for the perovskite solution. Table S1 summarizes the effect of DMF/DMSO mixture solvent on the performances of d-MAPI-HEA solar cells. The control cell, with pure DMF solvent, exhibited a poor performance of 5.33% with a low *J_SC_* of 10.21 mA.cm^−^^2^_._ The best cells were prepared for a mixture of 90/10 vol% DMF/DMSO. The champion cell delivered a PCE of 9.54% with an improved *J_SC_* and *FF* at 15.23 mA.cm^−^^2^ and 68.62%, respectively. This result is in line with most of the results found in the literature about the importance of the DMF/DMSO solvent mixture [65,66,67]. However, despite the improvement in efficiency, hysteresis remained a crucial issue, with a hysteresis index (*HI*) value of 29.4%. We tested the addition of a mixture of potassium chloride KCl and ammonium chloride NH_4_Cl. For 3D perovskites, these two additives were proven to have a synergistic effect by Pauporté’s group: NH_4_Cl is beneficial for the perovskite crystallinity, grain size, and overall device performance, while KCl prevents pinholes and PbI_2_ formation (due to its better solubilization) and robustly passivates defects. [52] They have also reported that K^+^ has the ability to block iodide migration and suppress hysteresis. [52] Various molar percentages of KCl and a mixture of KCl and NH_4_Cl were tested and are reported as KCl5, KCl9, KCl13, and KCl5/NH4Cl30 in the rest of the article. The combined incorporation of 5 mol% KCl and 30% NH_4_Cl afforded a better quality of the perovskite film, as can be seen through XRD measurements (Figure S1a). An interesting feature of these mixed additives is the prevention of the formation of the (004) peak at 28.2° (Figure S1b), which has been shown detrimental to a high-quality film. [68] An enhanced absorbance in the 350–550 nm range was also observed for the KCl5/NH4Cl30 film (Figure S1c). The higher absorbance led to an improvement of *J_SC_* that induced an improvement in the global efficiency of the system. The addition of KCl and NH_4_Cl also greatly reduced the dispersion of the results (Figure S2). However, in spite of these improvements, hysteresis was still present in the *J-V* curves (above 20%). It means that, unlike the 3D perovskites, KCl cannot fully suppress hysteresis for the d-MAPI-HEA system. It can be assigned to the special structure on the d-HPs that cannot allow the full blocking of ionic migration or charging accumulation at the interfaces.

**Figure 2:**
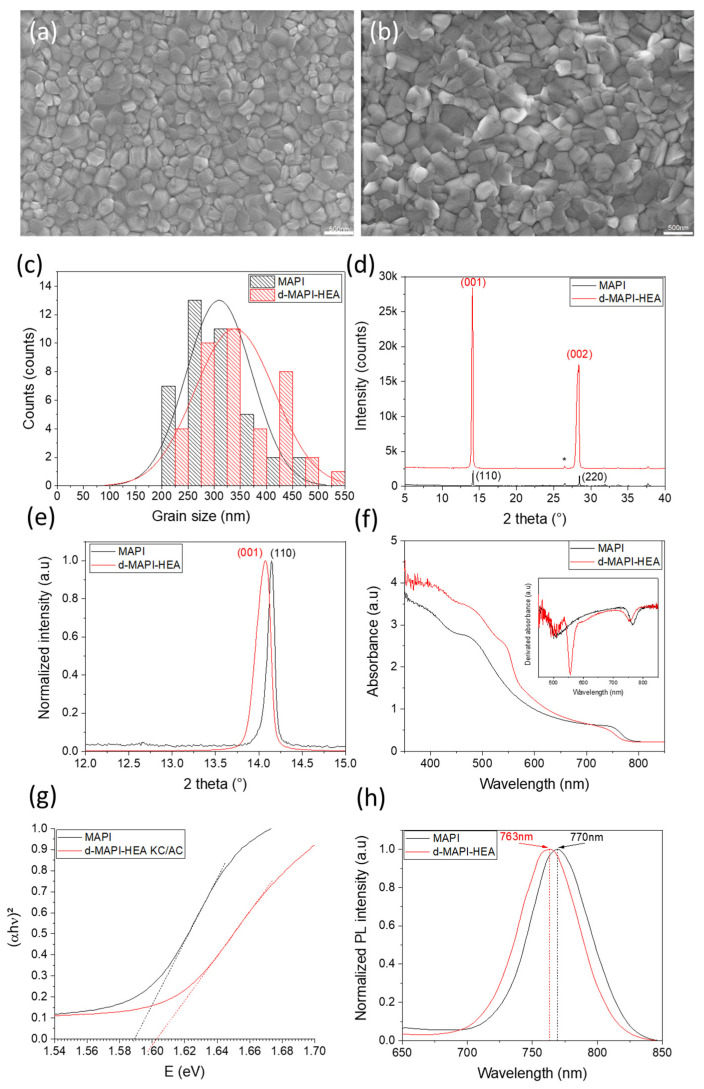
Top-view SEM images of (**a**) MAPI film and (**b**) d-MAPI-HEA film (Scale bar: 500 nm). (**c**) Grain size distribution in MAPI and d-MAPI-HEA films. (**d**) XRD patterns of MAPI and d-MAPI-HEA films (FTO peaks are indicated by the * symbol) (**e**) Same as (**d**), normalized and zoomed on (110) peak. (**f**) UV-Visible absorbance spectra (inset represents the derivative absorbances), (**g**) Tauc Plot, and (**h**) Steady-State PL of MAPI and d-MAPI-HEA perovskite films (λ_exc_ = 510 nm).

Morphological, structural, and optical properties of the final d-MAPI-HEA perovskite films were investigated and compared to classic MAPI perovskite. Figure 2a,b displays scanning electron microscopy (SEM) top-view images of MAPI and d-MAPI-HEA films, respectively. Both films presented a compact and pinhole-free morphology with perfect coverage and good grain size distribution (Figure 2c). Their average values are 310 nm and 350 nm for MAPI and d-MAPI-HEA, respectively. The grain size is slightly increased for d-MAPI-HEA_._ Cross-sectional images are displayed in Figure S3. The d-MAPI-HEA perovskite layer (Figure S3b) is dense and presents almost no defects (a small pinhole can be observed at the ETL/PVK interface), with a morphology very similar to the reference MAPI (Figure S3a) as was observed on top-view images. X-ray diffraction (XRD) was employed to check the phase and the purity of the produced perovskites. In Figure 2d, the layer of tetragonal MAPI is textured with two dominant XRD (110) and (220) reflection peaks. The XRD pattern of d-MAPI-HEA layers also presents two intense peaks indexed as (001) and (002), based on the single crystal full structure determination carried out in [54]. The high intensity of these peaks shows a high crystallinity of the perovskite film along with a preferential orientation. The d-MAPI-HEA film also exhibits a shift in the Bragg reflections to smaller 2θ angles (Figure 2e), as described elsewhere [54]. This shift indicates an expansion of the unit cell caused by the HEA^+^ cation and, thus, a good insertion within the MAPbI_3_ lattice. Moreover, the observed shift corresponds well with the expected x = 0.13 composition, as shown in Figure S4a. We noted a very small Bragg reflection peak at 12.7° for MAPI due to traces of PbI_2_ (Figure S4b). The absence of Bragg reflection peaks below 13° for the d-MAPI-HEA perovskite layer is in line with our previous studies at this level of substitution (x = 0.13) (Figure S4b). [54] The absence of the PbI_2_ parasitic phase is also noted. It attests to the remarkable purity of d-MAPI-HEA perovskite film. In addition to XRD, UV-Visible absorbance and photoluminescence (PL) were conducted. d-MAPI-HEA exhibits a better absorbance in the UV-Vis range (Figure 2f). A better absorbance can be related to a greater thickness, but it seems very unlikely here, considering Figure 2b, or larger grains. Both MAPI and d-MAPI-HEA presented an absorption edge around 780 nm caused by the direct optical transition in the metal-organic perovskite material. A new absorbance edge at 550 nm for d-MAPI-HEA was clearly visible on the absorbance derivative (Figure 2f, inset). Such an edge has been found on powders and layers at high x [69,70,71]. The presence of this edge indicates domains with high concentrations of PbI^+^ vacancies leading to a reduced bandgap. [54] We determined the main bandgap values by drawing Tauc Plots in Figure 2g. The estimated bandgaps are 1.587 eV and 1.602 eV for MAPI and d-MAPI-HEA, respectively, showing a slightly blue-shifted absorption for d-MAPI-HEA, coherent with the incorporation of HEA^+^ ions within the perovskite lattice. Steady-state photoluminescence spectra of the layers (Figure 2h) exhibit a blue shift of 7 nm of the PL peak for d-MAPI-HEA (770 nm for MAPI and 763 nm for d-MAPI-HEA). This blue shift is in agreement with the absorbance curves. It is important to note that the low substitution level of PbI^+^ by HEA^+^ (x = 0.13) results in a rather small change in the optoelectronic properties of the perovskite.

**Figure 3:**
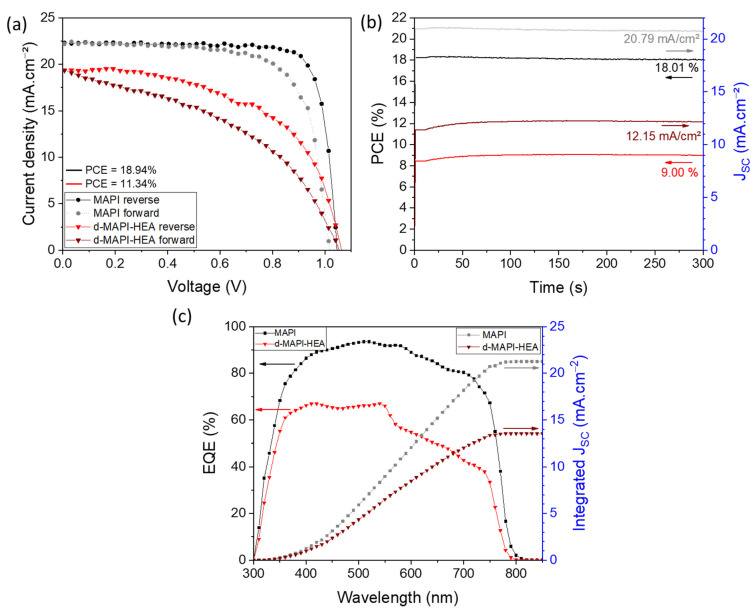
(**a**) *J-V* curves of best MAPI and d-MAPI-HEA devices. (**b**) Stabilized efficiency of best MAPI and d-MAPI-HEA devices (the applied voltages are 0.87 V and 0.74 V, respectively). (**c**) EQE spectra (left) with integrated *J_SC_* (right) of best MAPI and d-MAPI-HEA devices. The arrows point out the Y-axis to read.

Once the systems were fully characterized and optimized, we fabricated complete solar cells and measured their efficiencies (12 for each system). The d-MAPI-HEA system reached its best PCE at 11.47%, with *V_OC_* = 1.06 V, *J_SC_* = 19.36 mA.cm^−^^2^, and *FF* = 55.76%, measured on the reverse scan, and a hysteresis index of 23.1% (*PCE* = 8.82% on the forward scan). As a comparison, its classic analog MAPbI_3_ delivered a maximum efficiency of 18.94 %, with *V_OC_* = 1.04 V, *J_SC_* = 22.29 mA.cm^−^^2^, *FF* = 81.44%, and a hysteresis index of 14.5% (*PCE* = 16.20% on the forward scan) (Table S2, Supporting Information). Figure 3a displays the photocurrent density-voltage (*J-V*) curves of the representative cells. Data for the reverse and forward scans are gathered in Table 1. Associated box charts of each parameter of both MAPbI_3_ and d-MAPI-HEA cells are shown in Figure S5 (Supporting Information). An important reduction of the *J_SC_* is observed after HEA^+^ insertion within the MAPbI_3_ lattice, while the d-MAPI-HEA layer has better light absorption than the MAPI one (Figure 2f). This is mainly related to transport properties due to large cation insertion and charge accumulation at the interface with the selective contact (Figure S6 and Table S3). Stabilized *PCE* was measured by maximum power point (MPP) tracking (Figure 3b). The MAPI cell delivered a stabilized *PCE* of 18.01%, which is very close to the one measured on the reverse scan. On the other hand, the d-MAPI-HEA cell delivered a stabilized efficiency of 9.00%, which is in between the efficiency measured on the reverse and forward scans. External quantum efficiency (EQE) measurement was performed (Figure 3c) to determine the origin of the gap between stabilized *PCE* and the one measured on the reverse scan. A slight difference of onset is observed, around 810 nm for MAPI and 790 nm for d-MAPI-HEA, which is consistent with what is observed with the UV-Visible absorption shown in Figure 2f. Despite having a superior absorbance, the d-MAPI-HEA device exhibited a lower EQE than MAPI. The decrease in the EQE value is even more important for d-MAPI-HEA after 550 nm, which corresponds to the edge observed on UV-Visible spectra. The *J_SC_* values integrated from the EQE curves of MAPI and d-MAPI-HEA are 21.27 mA.cm^−^^2^ and 13.53 mA.cm^−^^2^, respectively. Regarding the MAPI cell, this value is in good agreement with the *J_SC_* extracted from the *J-V* curve. However, the *J_SC_* values for d-MAPI-HEA present an important mismatch. Important mismatches are often observed for systems that are recombination limited. Different hypotheses are made within the scientific community regarding the reason for this mismatch. Ref. [72] summarizes the main hypothesis. According to this article and regarding our system, the two main plausible hypotheses are: the ionic migration within the perovskite and the long-term degradation under long-term measurements. Nevertheless, as can be seen with the stabilized efficiency curves in Figure 3b, the d-MAPI-HEA system is stable under light measurement. Therefore, the reason why there is such an important mismatch for d-MAPI-HEA perovskite could be the ionic migration. Indeed, due to its distinctive structure that presents vacancies caused by the insertion of HEA^+^ big cation, we can suppose that in dHP, the ionic species (HEA^+^) would be able to migrate more easily through those vacancies, causing more important hysteresis and modifying dynamics simultaneously during long measurements such as EQE. 

**Figure 4:**
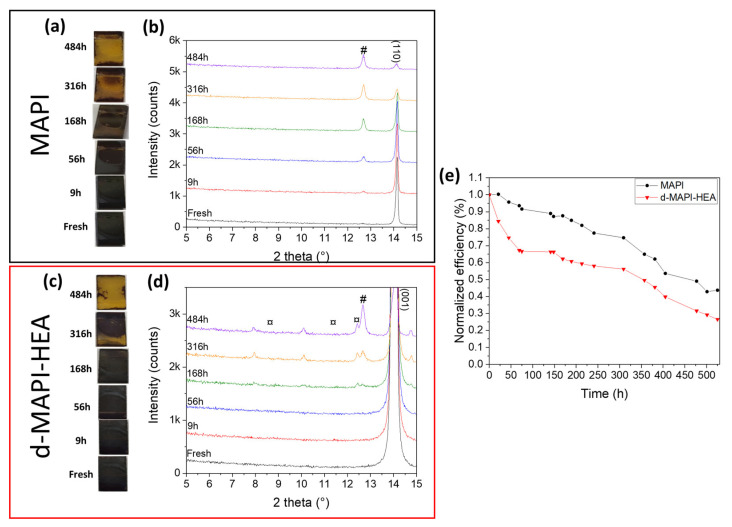
(**a**) Pictures of MAPI unencapsulated films upon aging. (**b**) XRD patterns upon the stability test of MAPI. (**c**) Pictures and (**d**) XRD patterns zoomed between 5° and 15° from the stability test of the unencapsulated d-MAPI-HEA films. FTO peaks are indicated by the ¤ symbol. # Indicates PbI_2_. ¤ symbol indicates unknown phases. (**e**) Evolution of normalized efficiency of unencapsulated MAPI and d-MAPI-HEA devices as a function of aging time. Storage upon aging tests was made in air under ambient conditions (50–70% RH; T° = 15–20 °C) and ambient light.

We then conducted stability tests. Complete unencapsulated cells and perovskite films were stored in air under ambient conditions (50–70% RH; T° = 15–20 °C) and ambient light. First, the aging of perovskite films was performed, followed by XRD measurements (Figure 4a and Figure S7). After only 9 h, the XRD pattern of MAPI film presented a small PbI_2_ at 12.6° that kept increasing in intensity until it reached its maximum after 484 h when complete degradation of MAPbI_3_ occurred. Contrastingly, d-MAPI-HEA started degrading into PbI_2_ after 168 h. After 316 h, new unknown peaks appeared at 7.9°, 10.1°, and 12.4°, but no indexation could be found in the literature, so we assume that those peaks are attributed to a new degraded phase of d-MAPI-HEA-like hydrate phases. For classic MAPI, monohydrate MAPbI_3_●H_2_O phases present diffraction peaks at 8.6° and 10.5°, and dihydrate (CH_3_NH_3_)_4_PbI_6_●2H_2_O present a peak at 11.4° [73]. After the apparition of these degradation products, aging was considerably accelerated, and after 484 h, the film became yellow, and (001) d-MAPI-HEA peak intensity at 14.1° dropped significantly but still remained more intense than the MAPI peak for the same aging time. This study demonstrates that HEAI incorporation greatly enhances the stability of the perovskite layer. Complete devices were aged to determine if d-MAPI-HEA PSCs are also more stable than the MAPI ones. Figure 4b displays the evolution of MAPI and d-MAPI-HEA cells under stability test. d-MAPI-HEA cell efficiency decreases faster than the MAPI one, with only 67% of its initial PCE retained after 75 h versus 91% for MAPI. For a longer aging time, this trend is no longer observed. The efficiencies of both systems decrease at the same pace to finally retain 34% and 45% of their initial *PCE* for d-MAPI-HEA and MAPI, respectively. In stark contrast, MAPI operational cells demonstrated better stability than d-MAPI-HEA despite a worse perovskite film stability. Then, HEA^+^ incorporation within the perovskite lattice increases its intrinsic stability, but the complete device stability is hindered.

### 2.2. d-FAPI-TEA Based Solar Cells

d-FAPI-TEA (x = 0.04) perovskite was reported in Ref. [74] and has never been tested in complete PSC. To efficiently develop d-FAPI-TEA PSCs, our attention focussed on a specific and peculiar solvent: the N-Methyl-2-Pyrrolidone (NMP). NMP is a known solvent for FAPbI_3_-based perovskite preparation. NMP has a high hydrogen-bond-accepting ability, which, combined with its more appropriate Lewis base properties, allows the formation of a much more stable FAI●PbI_2_●NMP adduct compared to DMSO [75]. The good nucleation of this more stable intermediate and also the rapid detachment of NMP during the annealing step induce the formation of a more uniform and covering film that ultimately cause a great reduction of defects [76,77,78]. According to the literature, the use of a small amount of NMP in combination with DMF in FAPbI_3_-based PSCs improved all *V_OC_*, *J_SC_*, and *FF* values, thus skyrocketing the efficiency of the devices [75,76,77,78,79,80,81,82]. We decided to test several DMF/NMP mixtures in a range of 0 to 30 vol% of NMP for d-FAPI-TEA preparation. Device efficiencies were measured and are given in Figure S8a–d. The best device parameters are reported in Table S4. Pure DMF-based cells exhibited a very low champion *PCE* of 2.68% with a high hysteresis. After the incorporation of NMP, a rise of the *PCE* was observed, up to 8.22%, for an optimized mixture of 80/20 volume ratio of DMF/NMP. This threefold increase was attained thanks to a simultaneous improvement of 0.12 V, 8.67 mA.cm^−2^, and 13.12% of *V_OC_*, *J_SC,_* and *FF,* respectively. After XRD and UV-Visible absorbance measurements (Figure S8e,f), no clear evidence of this enhancement was observed except for a moderately increased crystallinity. It is also important to note the absence of yellow/unwanted hexagonal δ-FAPI phase in the d-FAPI-TEA film with an annealing temperature of 125 °C which is below the one employed for annealing α-FAPI (153 °C). [83,84] SEM top images (Figure S9) revealed a compact and pinhole-free layer after incorporation of NMP, while pure-DMF film presented numerous cracks and pinholes hindering the cell efficiency. The substantial decrease in pinhole density resulted in reduced charge carrier recombination leading to an overall improved efficiency of working devices, as explained in the literature.

**Figure 5:**
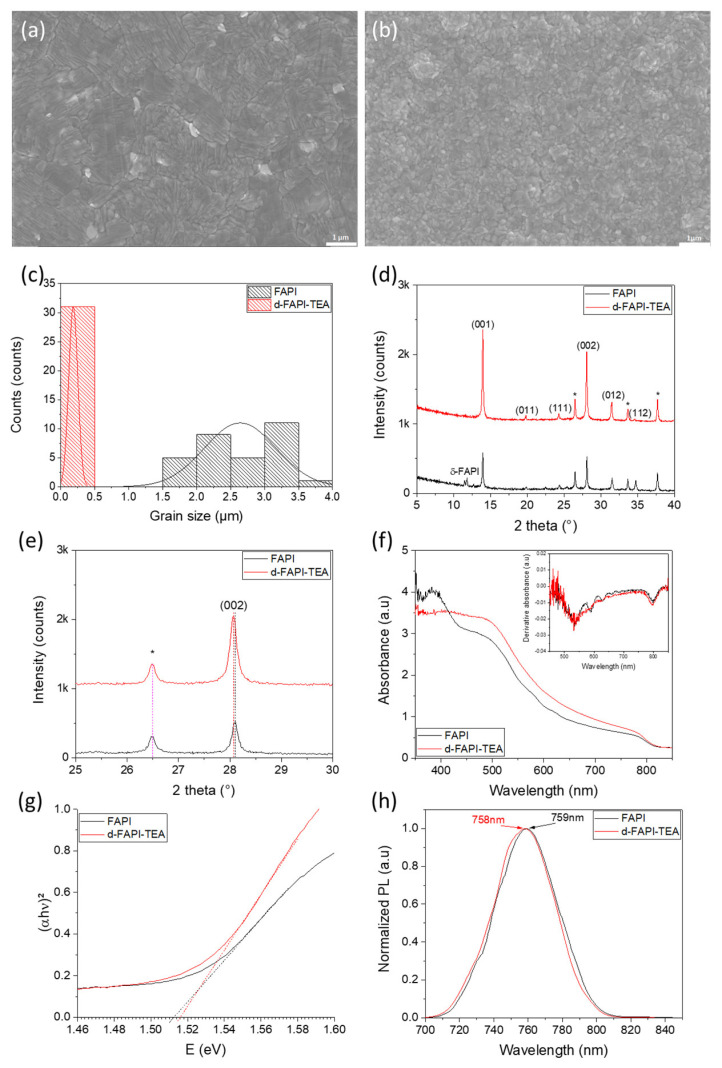
SEM images of (**a**) FAPI and (**b**) d-FAPI-TEA (x = 0.04) (Scale bar: 1 µm). (**c**) Grain size distribution of FAPI (large grains) and d-FAPI-TEA films. (**d**) XRD patterns of FAPI and d-FAPI-TEA films. (**e**) Same as (**d**) zoomed around (002) peak. FTO peaks are marked by the * symbol. (**f**) UV-Visible absorbance spectra of FAPI and d-FAPI-TEA perovskite films (the inset represents the derivative absorbances) (**g**) Tauc Plot of FAPI and d-FAPI-TEA absorption edge. (**h**) Steady-state photoluminescence (PL) of FAPI and d-FAPI-TEA films (λ_exc_ = 380 nm).

FAPI and d-FAPI-TEA samples were prepared as powder adducts washed with diethyl ether before being dried. Their differential Scanning Calorimetry (DSC) curves are shown in Figure S10. The FAPI curve displays endothermic peaks at 70–90 °C, 101.5 °C, and a large one with a minimum at 153.5 °C assigned to the crystallization of the black perovskite α-phase [85,86]. For d-FAPI-TEA perovskite, prepared with a DMF/NMP mixture with a volume ratio of 80/20, the shape is clearly different with a first broad peak observed at 50.9 °C and three others at 80.2 °C, 93.6 °C and 105.6 °C. In this case, the starting material contains the black perovskite α-phase. The two very different calorimetric behavior highlights that the two materials are different in nature. Scanning electron microscopy (SEM) was performed to assess the morphology of optimized d-FAPI-TEA perovskite and to compare it to its classic FAPbI_3_ counterpart. On top views (Figure 5a,b), both layers are covered and pinhole-free. FAPI film is made of highly textured micrometer-sized grains with PbI_2_ flakes (bright grains) dispersed throughout the surface. However, higher magnification images (Figure S11) show that the micrometer-sized grains seem to be polycrystalline, i.e., constituted of smaller grains. Unfortunately, these smaller grains are difficult to distinguish, so it is impossible to determine their size. On the other hand, d-FAPI-TEA film presents small grains around 200 nm in size (Figure 5c) without a trace of PbI_2_. Cross-sectional views of the films are shown in Figure S12. The two perovskites present completely different morphology. For the FAPI layer, we confirm the presence of large grains while no pinholes nor voids are present. The surface is not smooth. On the other hand, d-FAPI-TEA is made of smaller grains throughout its thickness which is detrimental for charge transport due to an increased number of grain boundaries. Moreover, voids could be observed near the ETL/PVK interface on this layer, which must greatly hinder the final efficiency of the complete devices. To complement the study of the morphology, we performed XRD measurements. Figure 5d shows the XRD pattern of the films. The addition of TEA^+^ increases the diffraction peak intensity of the film without changing its preferential orientation along the (001) and (002) plans. Higher peak intensity in the presence of TEA^+^ insertion is surprising in view of the poor morphological quality and small grains of these layers. Such a difference in crystallinity between FAPI and d-FAPI-TEA could also be caused by the not pure reference FAPI film (presence of yellow δ-FAPI phase) that hinders its crystallinity and then reduces the intensity of the peaks measured for this perovskite. Another noteworthy point is the total absence of δ-FAPI in the d-FAPI-TEA film, which is annealed at only 125 °C. On the other hand, FAPI films, annealed at 153 °C, present the δ-FAPI phase. Similarly to d-MAPI-HEA, d-FAPI-TEA exhibits a small shift of the (002) peaks to smaller 2θ values (Figure 5e), which is assigned to the good insertion of TEA^+^ cations within the FAPI perovskite lattice. The shift is yet less important because a smaller amount was employed for TEA^+^ compared to HEA^+^, mentioned earlier in the article. The total absence of peaks below 10° (Figure S13) is expected for the formation of pure-3D perovskite after small TEA^+^ incorporation. The UV-Visible absorbance spectra of the perovskite layers were measured (Figure 5f). d-FAPI-TEA displays a slightly better absorbance between 350 and 700 nm. By looking at the derivative absorbance, no additional peaks are observed for d-FAPI-TEA, which proves the obtaining of pure material with no undesired phases within the layer. On the other hand, the FAPI film presents several waves on its absorbance between 550 nm and 700 nm and the corresponding small peaks on its derivative absorbance spectrum. They can be assigned to the presence of impurity phases, such as yellow δ-FAPI detected by XRD measurements (Figure 5e) and interference fringes. Tauc plots were drawn to determine the optical bandgap value (E_g_) and see its evolution after TEA^+^ cation incorporation within the FAPbI_3_ lattice (Figure 5g). An increase of 6 meV in the bandgap was noticed for d-FAPI-TEA compared to FAPI, which is in agreement with the lattice expansion caused by the TEA^+^ insertion observed by XRD. Steady-state photoluminescence characterizations were conducted to complete UV-Visible measurements (Figure 5h). A small shift of 1 nm of the PL peak was observed after TEA^+^ incorporation (from 759 to 758 nm), surely due to the small amount inserted (4%).

**Figure 6:**
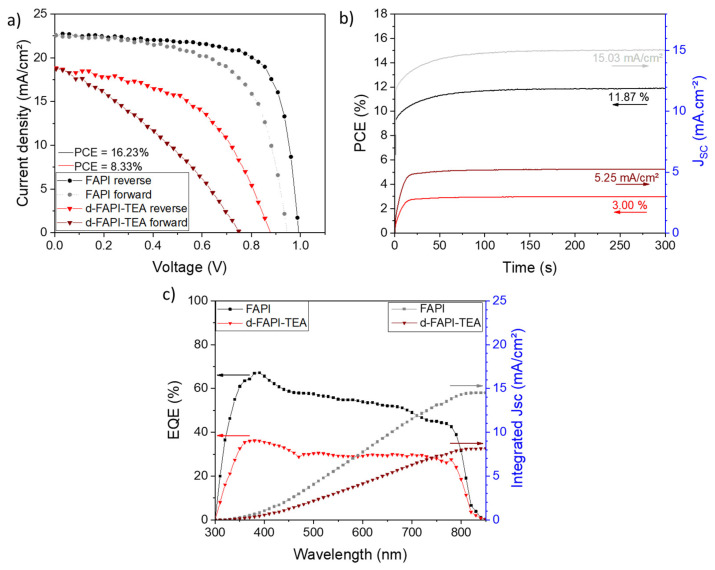
(**a**) *J-V* curves of best FAPI and d-FAPI-TEA devices. (**b**) Stabilized efficiency of best FAPI and d-FAPI-TEA devices (the applied *V_max_* are 0.79 V and 0.57 V, respectively). (**c**) EQE spectra (left) with integrated *J_SC_* (right) of best FAPI and d-FAPI-TEA devices. The arrows point out the Y-axis to read.

Ten complete devices were fabricated for each system, and their *J-V* curves were measured. On the one hand, FAPI delivered a maximum *PCE* of 16.23% (13.33% in forward scan), with a *V_OC_* = 0.99 V, *J_SC_* = 22.63 mA.cm^−^^2^, and *FF* = 72.40% when measured in the reverse scan, and a hysteresis of 17.9% (Table S5). Such efficiencies are in line with what can be found in the literature [76,80]. The hysteresis is comparable to the one we obtained for reference MAPI devices (Table S2) and is expected for simple perovskite without particular additives or post-treatment. On the other hand, d-FAPI-TEA delivered a maximum efficiency of 8.33% (4.71% in forward scan), with a *V_OC_* = 0.88 V, *J_SC_* = 18.68 mA.cm^−^^2^, and *FF* = 50.79% when measured in the reverse scan, and a hysteresis of 43.4% (Table S5). *J-V* curves of these cells are shown in Figure 6a. Data from reverse and forward scans are displayed in Table 2, and the corresponding box charts of both systems are shown in Figure S14. Stabilized efficiency of the best cells was measured (Figure 6g). Abnormally low efficiencies of 11.87% and 3.00% were obtained for FAPI and d-FAPI-TEA devices, respectively. EQE measurements were also conducted (Figure 6h) to understand these results. Spectra below our expectations were obtained. Indeed, for both systems, a low EQE curve with highly underestimated integrated *J_SC_* was found. The low values obtained for FAPI could be explained by its extremely poor stability, making the measured PSCs already slightly deteriorated between the *J-V* curves, the stabilized efficiency, and EQE measurements. Concerning d-FAPI-TEA, we can suppose that it is caused by the ionic migration and the presence of voids that is deleterious for charge transfer.

**Figure 7:**
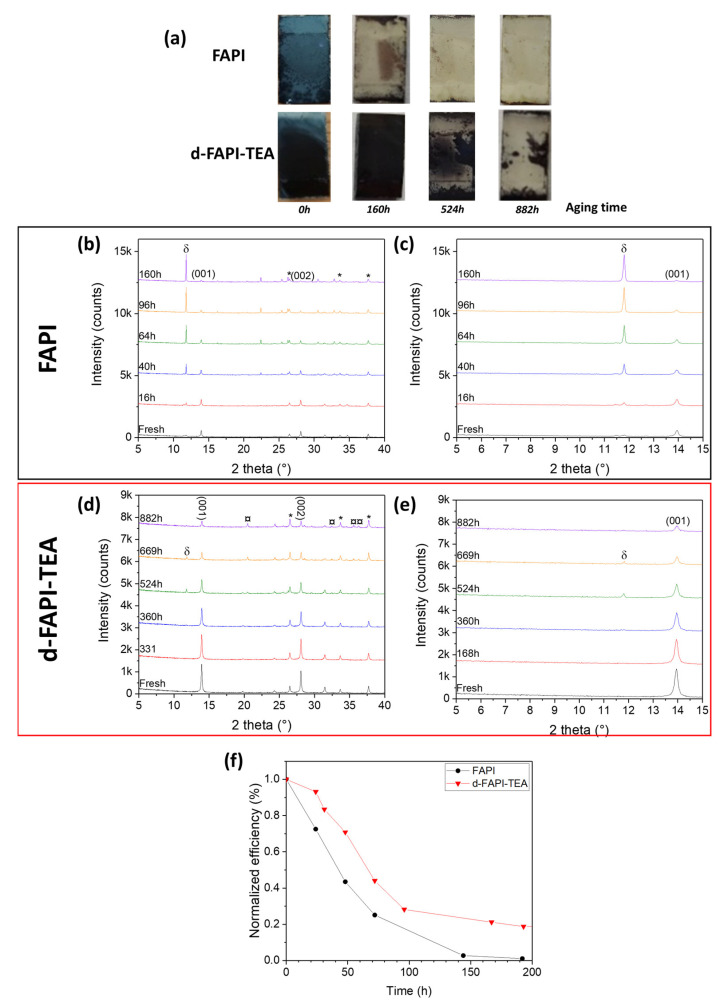
(**a**) Evolution of FAPI and d-FAPI-TEA films aspect upon aging (50–70% RH, T°=15–20°C, ambient light). (**b**) Complete XRD patterns recorded upon the stability test of FAPI films. (**c**) Same as (**b**) zoomed between 5–15°. FTO peaks are indicated by the * symbol. δ indicates yellow hexagonal δ-FAPI phase. (**d**) Complete XRD patterns recorded upon the stability test of d-FAPI-TEA films. (**e**) Same (**d**) zoomed between 5–15°. FTO peaks are indicated by the * symbol. δ indicates yellow hexagonal δ-FAPI phase. ¤ symbol indicates Pb(OH)I phase. (**f**) Evolution of normalized PCE unencapsulated FAPI and d-FAPI-TEA devices. Storage was made in air under ambient conditions (50–70% RH; T° = 15–20 °C) and ambient light.

Finally, we conducted stability tests on perovskite films and complete cells by storing them in ambient air (RH = 50–70%; T° = 15–20 °C). Figure 7a shows pictures of FAPI and d-FAPI-TEA films upon aging. Figure 7b,c depicts the XRD patterns over a large 2θ range (left panels) and zoomed between 5° and 15° (right panels) for FAPI and d-FAPI-TEA films, respectively. The XRD study shows that the transformation of α-FAPI to yellow δ-FAPI phase started after 16 h for FAPI and that it continued until the total disappearance of black α-FAPI after 160 h (in accordance with Figure 7a). This result is consistent with the low stability observed for FAPI cells. Concerning d-FAPI-TEA films, after 331 h, the film was not deteriorated, which makes d-FAPI-TEA perovskite film much more stable than the FAPI one. The intensity of the (001) peak started to decrease slightly after 360 h. Once 524 h (22 days) passed, degradation of α-d-FAPI-TEA into δ-d-FAPI-TEA began. After 669 h, the (001) perovskite peak was greatly reduced, but the δ-d-FAPI-TEA peak did not increase in intensity, while new peaks appeared at 20.5°, 32.4°, 35.6° and 36.2° (Figure 7c), that we assign to Pb(OH)I phase formation, resulting in a pale white appearance of the film. [87] This compound, formed after the inclusion of hydroxide anions within the structure after a reaction between the perovskite and the humidity, was not observed for classic FAPI. After 882 h, no more δ-d-FAPI-TEA was present in the film and only a small amount of α-d-FAPI-TEA and Pb(OH)I was left. Not only the incorporation of TEA^+^ within FAPI perovskite improved the stability of the film greatly, as can be seen in Figure 7a, but it also changed its degradation pathway by converting α-compound into Pb(OH)I.

The same stability test was performed on complete unencapsulated PSCs (Figure 7d). FAPI cells displayed a rapid drop in efficiency by retaining only 43% of their original PCE after only 48 h. After 6 days, the cell retained only 2% of its original *PCE*, going in short-circuit after 8 days. On the other hand, d-FAPI-TEA preserved 71% of its *PCE* after 48 h. After 8 days, the cell maintained 20% of its original *PCE,* where the FAPI device was short-circuited, which is a great improvement compared to FAPI.

## 3. Conclusions

In summary, we successfully developed d-MAPI-HEA (x = 0.13) and d-FAPI-TEA (x = 0.04) thin films and perovskite solar cells through various optimizations. XRD measurements confirmed the good insertion of HEA^+^ and TEA^+^ into the lattice with a shift to the lower angles of the (110) MAPI peak and (001) FAPI peak, respectively, an insertion that was double-checked by PL measurement with the blue shift of the PL peak. The resulting d-MAPI-HEA perovskite film was compact, pinhole-free, and very similar to its MAPbI_3_ peer with bigger grains. They also displayed better crystallinity and absorbance than MAPI. Overall, the PCE of d-MAPI-HEA devices reached 11.47%, with an average of 10.39%. This perovskite has been proven to be much more stable than its classic analog MAPI, which starts to degrade after only 9 h, whereas d-MAPI-HEA degradation begins after only 168 h. However, this significantly improved stability could not be reflected upon complete working PSCs.

On the other hand, d-FAPI-TEA final perovskite solar cells delivered a PCE of 8.33%. The optimized perovskite films were nonetheless not compact, made of small grains around 200 nm in size, and presented voids in their bottom. The insertion of TEA^+^ impressively improved the stability of the perovskite film since the degradation of d-FAPI-TEA into yellow δ-phase started only after 22 days in air, while classic FAPbI_3_ degradation began after 16 h and was total after 6 days. Unfortunately, this greater stability was not completely reflected in the device’s efficiency retaining. It is extremely important to note that TEA^+^ insertion within the FAPbI_3_ perovskite lattice allows the formation of stable black α-phase for an annealing temperature of 125 °C, which is below the commonly used 150 °C and higher temperatures.

## 4. Experimental Methods

### 4.1. Preparation of Substrate, Compact TiO_2_, and Mesoporous TiO_2_ Layers

Fluorine-doped SnO_2_ (FTO) substrates (TEC 7 from Pilkington) were etched pattern by zinc oxide powder and 10% HCl solution prior to being cleaned with soap and water. The substrates were subsequently plunged for 20 min in a concentrated 2.2 m NaOH in ethanol/water (10:1 volume ratio) and followed by cleaning in acetone using an ultrasonic bath for 12 min, then rinsed with deionized water in an ultrasonic bath for 15 min. [52] The substrates were subsequently heated at 500 °C for 15 min. The compact TiO_2_ electron transporting layer (ETL), noted c-TiO_2_, was prepared by aerosol spray pyrolysis. The mesoporous TiO_2_ ETL, noted m-TiO_2_, was prepared using a nanoparticle solution made in advance and stirred for at least 12 h. The anatase TiO_2_ NR30-D paste (from Greatcell Solar Materials, Queanbeyan, Australia) was diluted in ethanol with a 1:7 *w*/*w* ratio. An amount of 45 μL of the solution was dropped on the compact TiO_2_ layer and spin-coated at 2000 rpm for 15 s. The layer was then dried on a hotplate at 70 °C for at least 10 min and finally heated at 500 °C under an air flux for 30 min, cooled down to 200 °C and removed from the hotplate before being transferred immediately to an N_2_-filled glovebox for perovskite layer deposition.

### 4.2. Preparation of the Perovskite Layers

MAPbI_3_ perovskite. A perovskite precursor solution with a concentration of 1.35 m was prepared by mixing 214.6 mg of MAI (Greatcell Solar Materials) and 622.3 mg of PbI_2_ (TCI) in 1 mL of DMSO. The bottle was tightly capped. The solution was stirred for 2 h at 100 °C in an N_2_-filled glovebox. An amount of 40 µL of this solution was placed on top of the substrate before starting the spin-coating in an N_2_-filled glovebox. The program used was: 1000 rpm with an acceleration of 200 rpm/s for 10 s followed by a second spinning at 6000 rpm with an acceleration of 4000 rpm/s for 30 s. Moreover, 100 µL of chlorobenzene was dripped 30 s after the start of the spinning routine at a slow speed using a manual micropipette (put at an angle of 25–30° to the horizontal and at a distance of 5–7 mm from the substrate). The films were then annealed on a hotplate at 105 °C for 1h inside the N_2_-filled glovebox.

d-MAPI-HEA (x = 0.13) perovskite. A perovskite precursor solution with a concentration of 1 m was prepared by mixing 59.7 mg of HEAI (prepared as described in Ref. [54]), 143 mg of MAI (Greatcell Solar Materials), 461 mg of PbI_2_ (TCI), 3.7 mg of KCl (Alfa Aesar) and 15 mg NH_4_Cl (Alfa Aesar) in a mixture of 900 µL of DMF and 100 µL of DMSO. The solution was stirred for 4 h at 50 °C in an N_2_-filled glovebox. Then, 40 µL of this solution was deposited on top of the substrate before starting the spin-coating. A two-step spin-coating program was used: first spinning at 1000 rpm with an acceleration of 500 rpm/s for 10 s, followed by a second spinning at 5000 rpm with an acceleration of 1500 rpm/s for 30 s. For the quenching, 100 µL of chlorobenzene was dripped 15 s after the start of the spinning routine using an electronic micropipette Eppendorf Xplorer with a defined and optimized ejection speed of v = 3 (put at an angle of 25–30° to the horizontal and at a distance of 5–7 mm from the substrate) to optimize as much as possible the reproducibility. The films were finally annealed at 105 °C for 1 h.

FAPbI3 perovskite. A perovskite precursor solution with a concentration of 1.2 m was prepared by mixing 206 mg of FAI (Greatcell Solar Materials) and 553 mg of PbI_2_ (TCI) in a solvent mixture of 800 µL DMF and 200 µL DMSO. The solution was stirred for 2 h at room temperature in an N_2_-filled glovebox. About 40 µL of this solution was placed on top of the substrate before starting the spin-coating. A two-step spin-coating program was used: first, spinning at 1000 rpm with an acceleration of 1000 rpm/s for 10 s to homogeneously spread the solution. Then the spinning was raised to 6000 rpm with an acceleration rate of 4000 rpm/s for 30 s. An amount of 100 µL of chlorobenzene was dripped 20 s after the start of the spinning routine using an electronic micropipette Eppendorf Xplorer with a defined and optimized ejection speed set at v = 3 (put at an angle of 25–30° to the horizontal and at a distance of 5–7 mm from the substrate) to optimize as much as possible the reproducibility. The films were then annealed at 153 °C for 13 min [88].

d-FAPI-TEA (x = 0.04) perovskite. Prior to perovskite deposition, a self-assembled monolayer (SAM) of 4-Chlorobenzoic Acid was deposited onto m-TiO_2_. [1] A perovskite precursor solution with a concentration of 1.1 m was prepared by mixing 37.6 mg of TEAI (prepared as described in Ref. [55]), 189.1 mg of FAI (Greatcell Solar Materials) and 507 mg of PbI_2_ (TCI) in a solvent mixture of 800 µL DMF and 200 µL NMP. The solution was stirred for 4 h at 50 °C in an N_2_-filled glovebox. About 40 µL of this solution was deposited on top of the substrate before starting the spin-coating. A two-step spin-coating program was used: first spinning at 1000 rpm for 10 s with an acceleration of 200 rpm/s followed by a second spinning at 6000 rpm for 20 s with an acceleration of 4000 rpm/s. 100 µL of chlorobenzene was dripped 15 s after the start of the spinning routine using an electronic micropipette Xplorer (Eppendorf, Montesson, France) with a defined and optimized ejection speed set at v = 3 (put at an angle of 25–30° to the horizontal and at a distance of 5–7 mm from the substrate) to optimize as much as possible the reproducibility. The films were then annealed at 125 °C for 30 min.

### 4.3. Preparation of Spiro-OMeTAD (HTM) Layer and Gold Back Electrode

The hole transporting layer (HTL) solution was prepared by dissolving 78 mg of spiro-OMeTAD (Borun New Material Technology) in 1 mL of chlorobenzene. Then, 17.9 μL of bis(trifluoromethylsulfonyl)imide lithium salt solution (Li-TFSI) (Sigma-Aldrich Chimie, Saint-Quentin Fallavier, France) solution (517 mg in 1 mL ACN), 30.4 μL of TBP (tert-butylpyridine) (Sigma-Aldrich Chimie, Saint-Quentin Fallavier, France), and 14 μL of tris(2–1H-pyrazol-1-yl)-4-tert-butylpyridine)-cobalt(III) tri(bis(trifluoromethylsulfonyl)imide), FK209 (from Greatcell Solar Materials, Queanbeyan, Australia) (376 mg in 1 mL acetonitrile) was added to this solution. About 40 μL of the HTM solution was spin-coated at 4000 rpm for 30 s. Finally, a back-electrode was deposited by thermally evaporating a 70–80 nm-thick gold layer on the spiro-OMeTAD layer.

### 4.4. Layers and Devices Characterizations 

Perovskite film structure was characterized using a PANanalytical X-Pert high-resolution XRD operated at 40 kV and 45 mA and using the Cu Kα radiation with λ = 1.5406 Å. The film specular absorbance was measured by a Cary 5000 UV–vis–NIR spectrophotometer using an integrating sphere. A glass/FTO/c-TiO_2_/m-TiO_2_ sample was used for the baseline. Steady-state photoluminescence (PL) was measured by a Cary Eclipse fluorescence spectrophotometer using an excitation wavelength of 510 nm for MAPI-based perovskites and 380 nm for FAPI-based perovskites. The morphology of PVK thin films was measured using field-emission SEM equipment (Zeiss Leo 40, manufacturer, city, state abbreviation, country) in the in-lens mode.

The solar cells’ *J-V* curves were recorded by a Keithley 2410 digital source meter using a 0.1 V.s^−1^ voltage scan rate. The solar cells were illuminated with a solar simulator Sun 2000 (Abet Technology, Villeurbanne, France) filtered to mimic AM 1.5G conditions (100 mW cm^−2^). [72] The illuminated surface was delimited by a black mask with an aperture diameter of 3 mm. The power density was calibrated at 100 mW cm^−2^ by the use of a reference silicon solar cell. The maximum power tracking measurements (MPP) were performed under ambient conditions similar to *J-V* measurement conditions. The current was followed at the voltage value comprised between the maximum power of reverse and forward scans. The external quantum efficiency (EQE) was measured using aQUANTX-300 system (Oriel, Évry, France). The light beam was chopped at 25 Hz. The monochromatic illumination was calibrated by a NIST-calibrated Si photodiode. Differential Scanning Calorimetry (DSC) curves were measured on precipitated sample adducts by a DSC 3 apparatus from the STAR system (Mettler Toledo, Schwerzenbach, Switzerland) operated under an N_2_ atmosphere with a heating rate of 10 °C/min.

## Figures and Tables

**Table 1:** Average photovoltaic *J-V* parameters with standard deviation of MAPI and optimized d-MAPI-HEA (x = 0.13).

Sample	Scan Direction	V_OC_ [V]	J_SC_ [mA.cm^−^^2^]	FF [%]	PCE [%]	HI [%] ^a)^
MAPI	Reverse	1.05 ± 0.01	22.29 ± 0.49	78.74 ± 1.93	18.46 ± 0.33	15.1 ± 3.3
	Forward	1.00 ± 0.02	22.28 ± 0.41	70.02 ± 2.30	15.68 ± 0.78	
d-MAPI-HEA	Reverse	1.04 ± 0.02	16.61 ± 1.78	60.14 ± 2.28	10.39 ± 0.77	24.6 ± 5.4
	Forward	1.00 ± 0.04	17.13 ± 1.90	45.90 ± 5.41	7.82 ± 0.63	

^a)^ Hysteresis index, noted HI, defined as (PCE_rev_−PCE_fwd_) × 100/PCE_rev._

**Table 2:** Average photovoltaic *J-V* curve parameters, *PCE,* and *HI* with standard deviation of FAPI and d-FAPI-TEA.

Sample	Scan Direction	*V_OC_* [V]	*J_SC_* [mA.cm^−^^2^]	*FF* [%]	*PCE* [%]	*HI* [%]
FAPI	Reverse	0.99 ± 0.01	21.74 ± 0.63	69.74 ± 1.38	15.02 ± 0.60	19.9 ± 4.0
	Forward	0.94 ± 0.02	21.67 ± 0.68	58.77 ± 2.25	12.04 ± 0.94	
d-FAPI-TEA	Reverse	0.84 ± 0.05	15.91 ± 1.21	49.41 ± 1.34	6.61 ± 0.86	54.2 ± 7.7
	Forward	0.73 ± 0.05	15.47 ± 1.51	26.71 ± 3.96	3.04 ± 0.77	

## Data Availability

The data presented in this study are available on request from the corresponding author.

## Supplementary Materials

The following supporting information can be downloaded at: https://www.mdpi.com/article/10.3390/nano13071245/s1, Figure S1: Effects of KCl additive and KCl/NH_4_Cl co-additives on: (**a**) XRD pattern of d-MAPI-HEA films; (**b**) Same as (**a**) zoomed around (200) peak; (**c**) UV-Visible absorbance spectra of d-MAPI-HEA films; (**d**) Derivative absorbance of d-MAPI-HEA films; Figure S2. Box plots of d-MAPI-HEA PSCs (**a**) *V_OC_* (**b**) *J_SC_* (c) *FF* (**d**) PCE for various molar percentages of KCl and NH_4_Cl; Figure S3. Cross-sectional SEM images of (**a**) MAPI, (**b**) d-MAPI-HEA. Scale bar: 500 nm; Figure S4. (**a**) PXRD patterns of the x=0.13 thin film (red) and an x= 0.13 crystallized powder sample extracted from the work reported in Ref.[54], showing that X-ray lines of these two x = 0.13 samples well-fit. (**b**) XRD patterns of MAPI and d-MAPI-HEA films zoomed between 5° and 13°. Figure S5. Box charts of MAPI and d-MAPI-HEA PSCs (**a**) *V_OC_* (**b**) *J_SC_* (**c**) *FF* (**d**) *PCE* (**e**) *HI* measured on the reverse scan; Figure S6. Normalized time-resolved photoluminescence of MAPI and d-MAPI-HEA films with and without additives. (**a**) is a zoom at a short time of (**b**). The full lines are the fit curves; Figure S7. XRD patterns from the stability test (50–70 % RH, T°= 15–20 °C) of MAPI (left) and d-MAPI-HEA (right) films. FTO peaks are indicated by the * symbol. # indicates PbI_2_. XRD corresponding film images upon their aging are displayed in the middle panel; Figure S8. Box plots of d-FAPI-TEA devices for various DMF/NMP ratios (**a**) *V_OC_*, (**b**) *J_SC_*, (**c**) *FF*, and (**d**) *PCE* on the reverse scan. The x-axis indicates the volume ratio of DMF/NMP. Effect of DMF/NMP solvent volume ratios on: (**e**) XRD pattern (* indicates FTO) and (**f**) UV-Visible absorbance curves of d-FAPI-TEA perovskite films for various DMF/NMP ratios; Figure S9. SEM images of d-FAPI-TEA layers prepared with (**a**) pure DMF solvent and (**b**) a mixture of 80DMF/20NMP solvents (vol%). Scale bar: 1 µm; Figure S10. DSC curves of (**a**) FAPI and (**b**) d-FAPI-TEA; Figure S11. SEM images of (**a**) FAPI (**b**) d-FAPI-TEA. Scale bar: 500 nm; Figure S12. Cross-section SEM images of (**a**) FAPI and (**b**) d-FAPI-TEA. Au electrode was present on top of d-FAPI-TEA film. Scale bar: 500 µm; Figure S13. XRD patterns of FAPI and d-FAPI-TEA films zoomed between 5°–13.5°; Figure S14. Box charts of FAPI and d-FAPI-TEA PSCs *J-V* curve parameters. (**a**) *V_OC_*; (**b**) *J_SC_*; (**c**) *FF*; (**d**) *PCE* measured on reverse scans; and (**e**) *HI.* Table S1. Efficiency of d-MAPI-HEA devices with different DMF/DMSO solvent ratio; Table S2. *J-V* parameters, *PCE*s, and *HI*s of best MAPI and d-MAPI-HEA optimized PSCs; Table S3. Exponential function used for TRPL and extracted parameters; Table S4. *J-V* curves parameters, *PCE*s and *HI*s of d-FAPI-TEA PSCs with different DMF/NMP volume ratios; Table S5. *J-V* parameters, *PCE* and *HI* of best FAPI and optimized d-FAPI-TEA PSCs.
