# Supplementary material for: Exploring Solar Cells Based on Lead- and Iodide-Deficient Halide Perovskite (d-HP) Thin Films
Source: Nanomaterials (Basel). 2023 Mar 31;13(7):1245. doi: 10.3390/nano13071245 (PMC10096836; doi:10.3390/nano13071245)
Supplement: Supplementary file 1 [file nanomaterials-13-01245-s001.zip › nanomaterials-2280301-SI.pdf]

nanomaterials

MDPI

Supplementary Materials

Exploring Solar Cells Based on Lead- and Iodide-Deficient Halide Perovskite (d-HP) Thin Films

Liam Gollino [1], Nicolas Mercier [2] and Thierry Pauporté [1,*]

[1] Institut de Recherche de Chimie-Paris (IRCP), UMR8247, CNRS, Chimie-ParisTech, PSL Université, 11 rue Pierre et Marie Curie, CEDEX 5, F-75231 Paris, France; liam.gollino@chimieparistech.psl.eu

[2] MOLTECH-Anjou, UMR 6200, University of Angers, 2 boulevard de Lavoisier, 49045 Angers, France; nicolas.mercier@univ-angers.fr

* Correspondence: thierry.pauporte@chimieparistech.psl.eu

Table S1. Efficiency of d-MAPI-HEA devices with different DMF/DMSO solvent ratio.

	Scan direction	V_{OC} [V]	J_{SC} [mA.cm^{-2}]	*FF* [%]	*PCE* [%]	*HI* [%] [a)]
DMF	Reverse	0.93	10.21	56.93	5.33	5.4
	Forward	0.97	9.90	53.23	5.04	
90 DMF/10 DMSO	Reverse	0.91	15.23	68.62	9.54	29.4
	Forward	0.84	13.31	59.95	6.74	
75 DMF/25 DMSO	Reverse	1.02	13.64	58.91	8.16	26.1
	Forward	1.01	12.65	47.43	6.03	
50 DMF/50 DMSO	Reverse	0.93	5.82	52.26	2.82	3.9
	Forward	0.91	5.70	52.27	2.71	

a) Hysteresis index, noted HI, defined as $(PCE_{rev}-PCE_{fwd})\times 100/PCE_{rev}$.

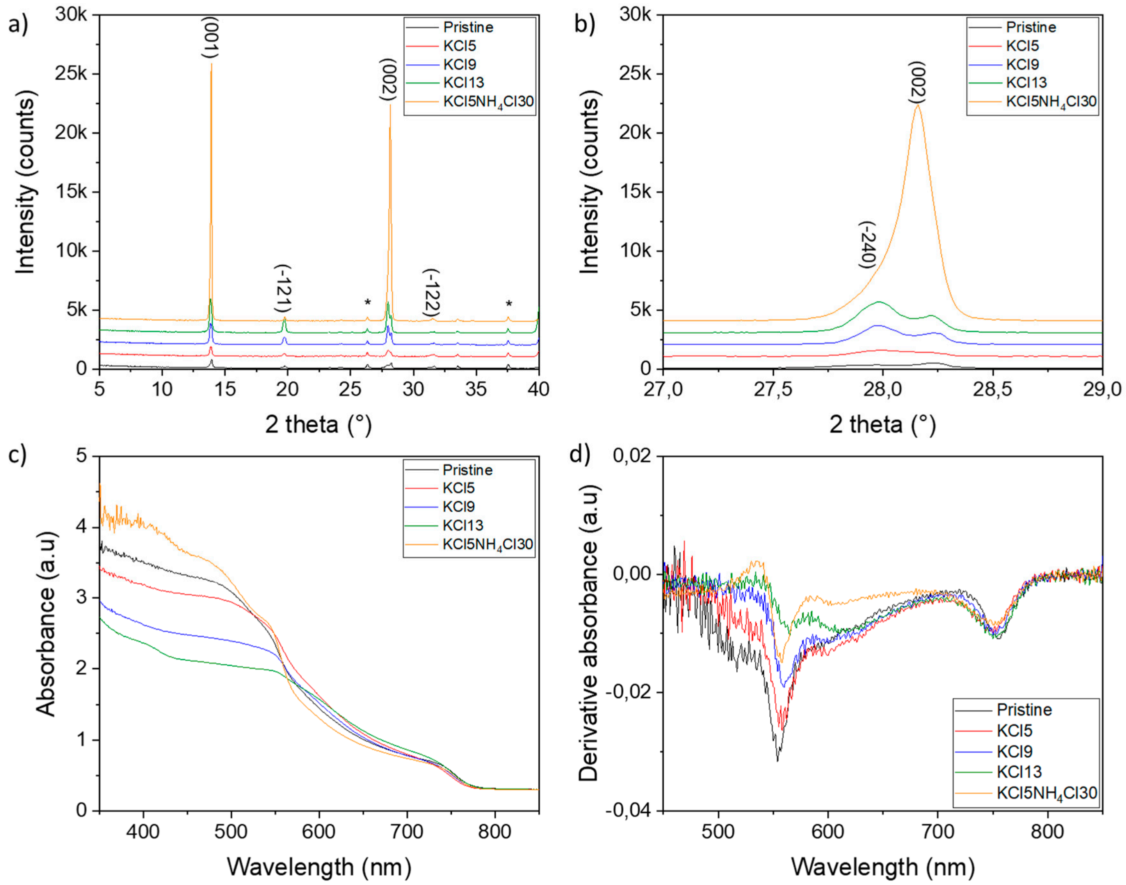

Figure S1. Effects of KCl additive and KCl/NH_4Cl co-additives on: (**a**) XRD pattern of d-MAPI-HEA films; (**b**) Same as (**a**) zoomed around (200) peak; (**c**) UV-Visible absorbance spectra of d-MAPI-HEA films; (**d**) Derivative absorbance of d-MAPI-HEA films.

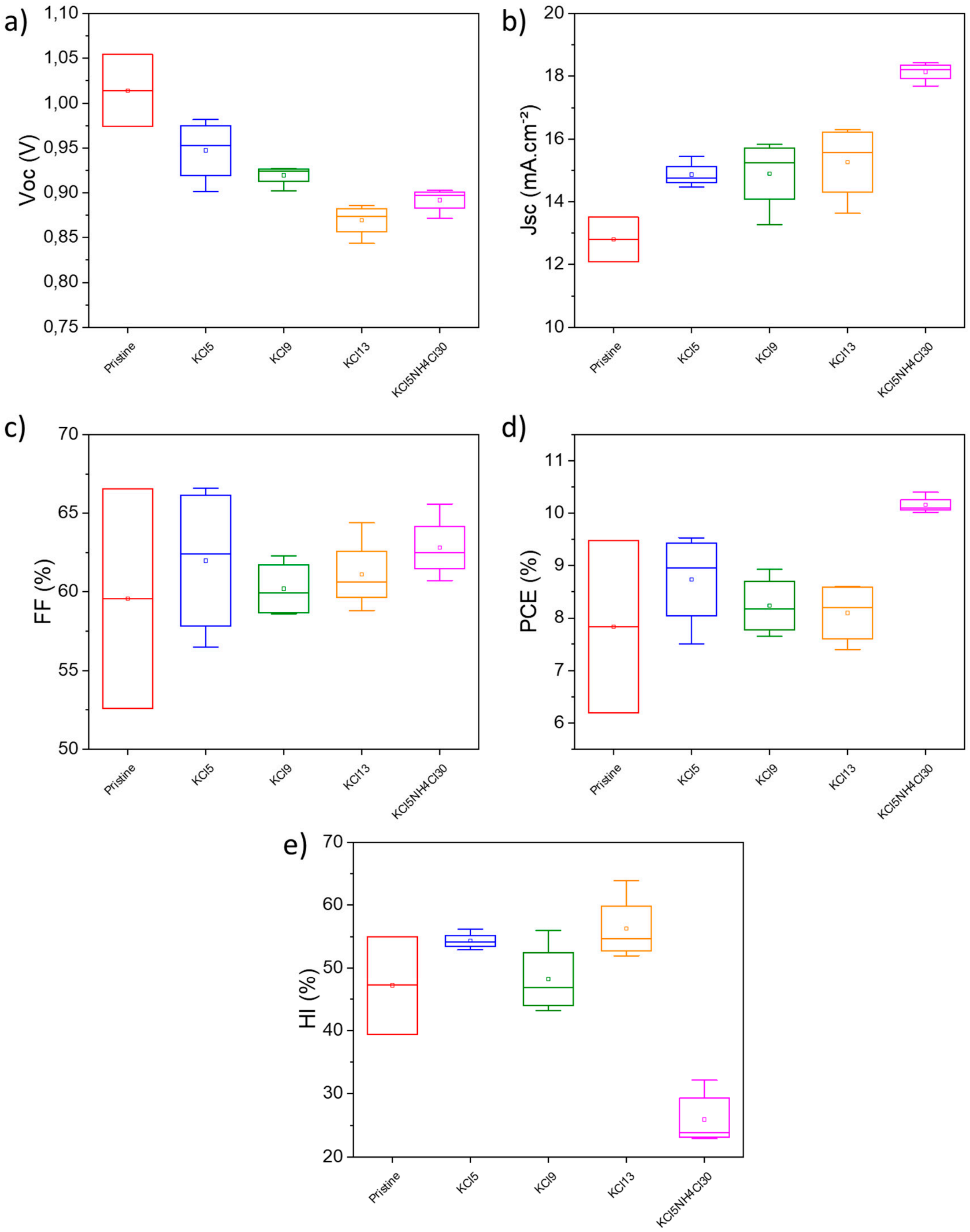

Figure S2. Box plots of d-MAPI-HEA PSCs (**a**) V_{OC} (**b**) J_{SC} (**c**) *FF* (**d**) *PCE* for various molar percentages of KCl and NH_4Cl.

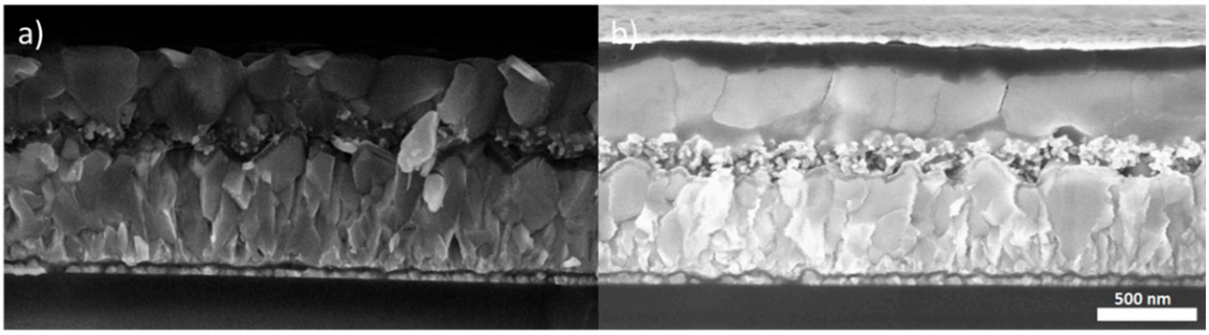

Figure S3. Cross-sectional SEM images of (**a**) MAPI, (**b**) d-MAPI-HEA. Scale bar: 500 nm.

(a)

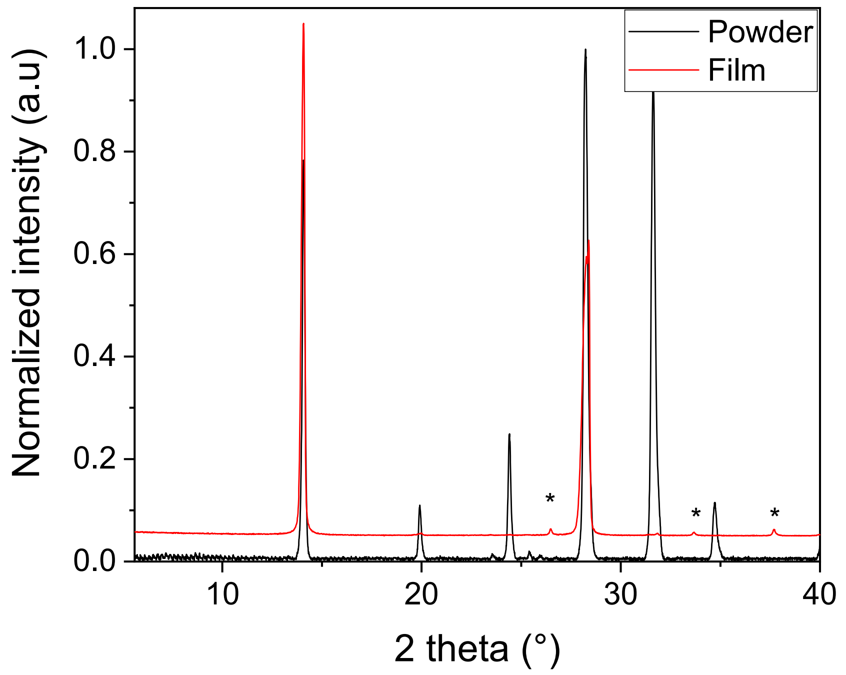

(b)

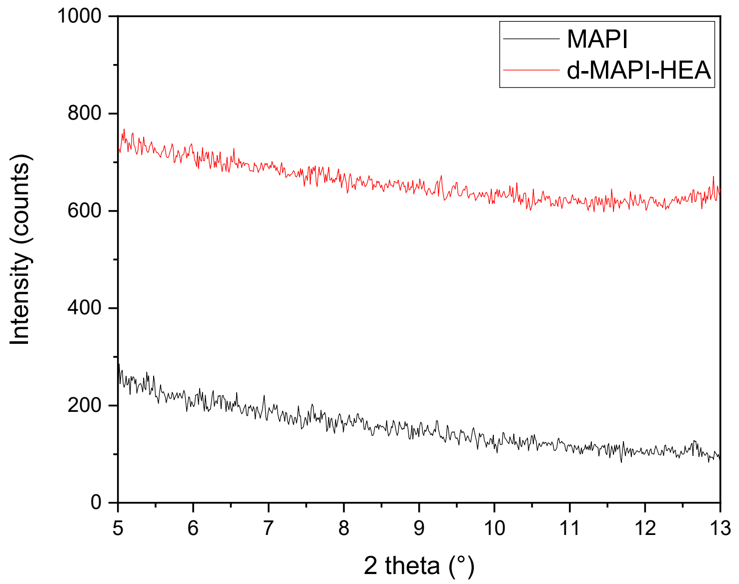

Figure S4. (**a**) PXRD patterns of the x=0.13 thin film (red) and an x= 0.13 crystallized powder sample extracted from the work reported in Ref.[54], showing that X-ray lines of these two x = 0.13 samples well-fit. Astericks indicate FTO lines. (**b**) XRD patterns of MAPI and d-MAPI-HEA films zoomed between 5° and 13°.

Table S2. *J-V* parameters, *PCE*s, and *HI*s of best MAPI and d-MAPI-HEA optimized PSCs.

Sample	Scan direction	V_{OC} [V]	J_{SC} [mA.cm^{-2}]	*FF* [%]	*PCE* [%]	*HI* [%]
MAPI	Reverse	1.04	22.29	81.44	18.94	14.5
	Forward	1.02	22.33	71.29	16.20	
d-MAPI-HEA	Reverse	1.06	19.36	55.76	11.47	23.1
	Forward	1.05	19.54	42.96	8.82	

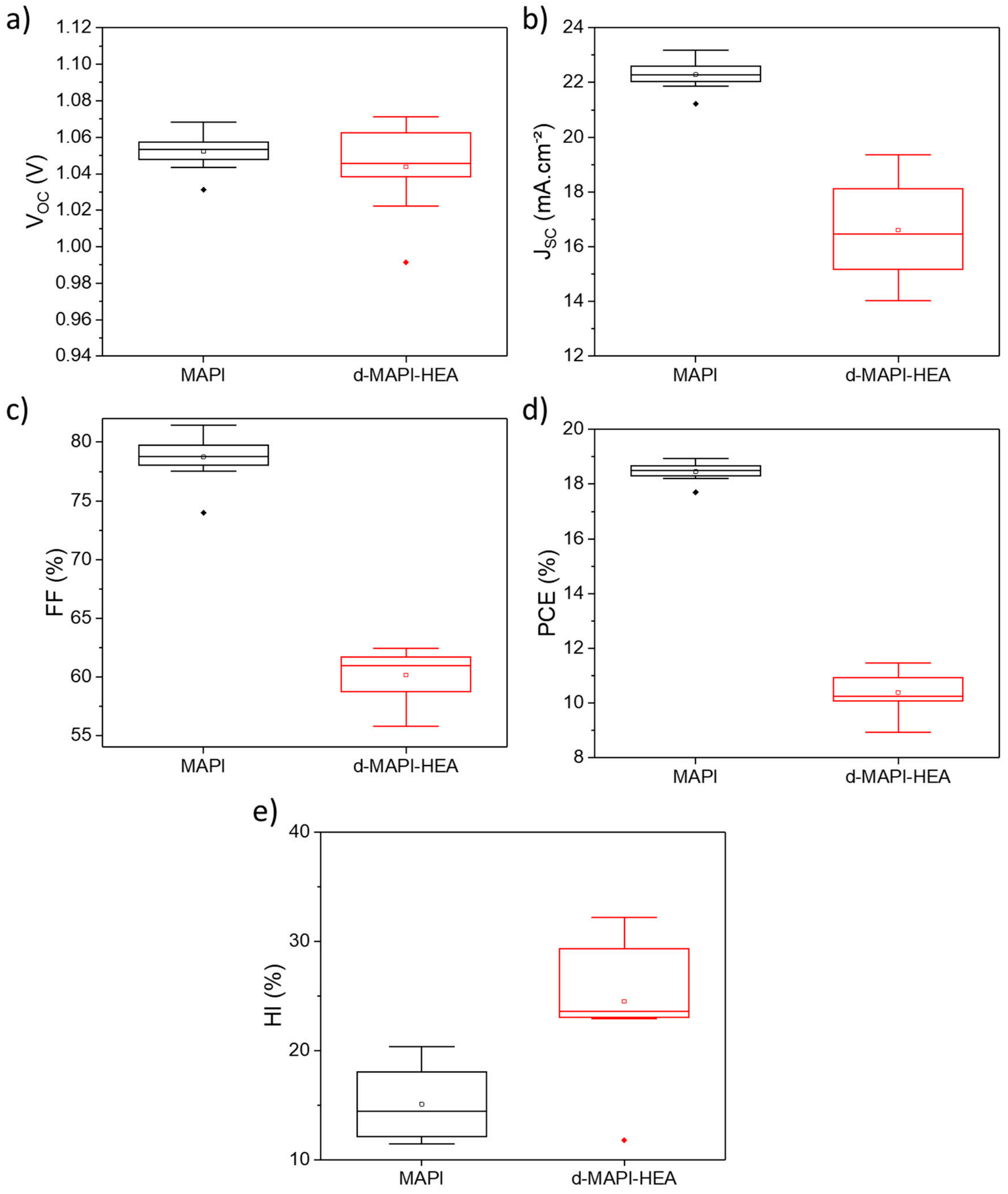

Figure S5. Box charts of MAPI and d-MAPI-HEA PSCs (**a**) V_{OC} (**b**) J_{SC} (**c**) *FF* (**d**) *PCE* (**e**) *HI* measured on the reverse scan.

Time-Resolved Photoluminescence (TRPL) Study of MAPI and d-MAPI-HEA Thin Films

The time-resolved photoluminescence (TRPL) measurements were performed under a microscope lens (numerical aperture 0.7). The perovskite layers were spin-coated onto a glass/FTO/c-TiO_2/m-TiO_2 substrate. The top of the PVK layer was excited by a 470 nm diode laser (Picoquant) filtered by a 488 nm long pass filter. The emission was analyzed for time-resolved photoluminescence (TRPL) decay by a PerkinElmer SPCM avalanche photodiode combined with a Picoharp acquisition card (500 ps characteristic time of the total system response function) used with the laser in a pulsed mode at a 10 nW excitation power (pulse duration 70 ps)

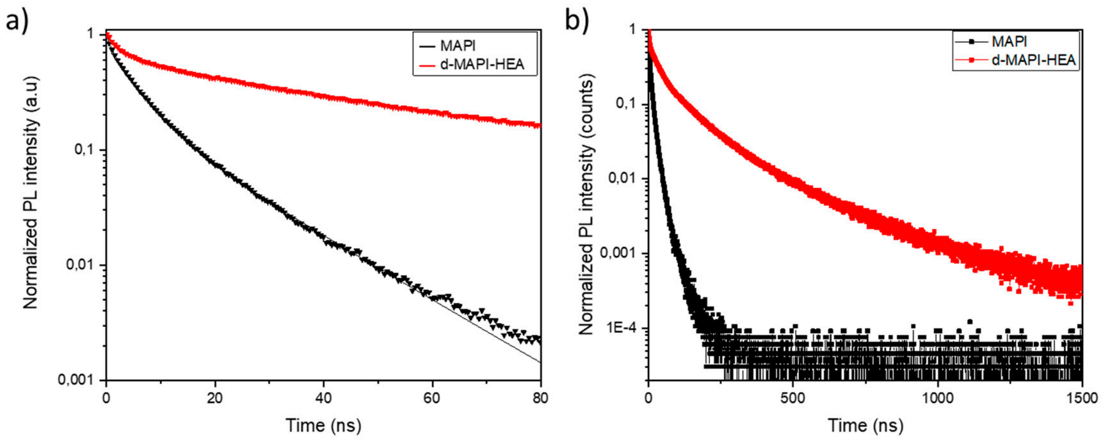

Figure S6. Normalized time-resolved photoluminescence of MAPI and d-MAPI-HEA films with and without additives. (**a**) is a zoom at a short time of (**b**). The full lines are the fit curves.

Table S3. Exponential function used for TRPL and extracted parameters.

**ExpDec3**: $y = y_0 + A_1 \exp\left(-\frac{t}{\tau_1}\right) + A_2 \exp\left(-\frac{t}{\tau_2}\right) + A_3 \exp\left(-\frac{t}{\tau_3}\right)$							
**Sample**	y_0	A_1	τ_{fast}	A_2	τ_{int}	A_3	τ_{slow}
MAPI	4.14E-5	0.19	0.72	0.58	5.00	0.23	15.62
d-MAPI-HEA	4.82E-4	0.36	2.60	0.43	32.86	0.21	148.43

Time-resolved PL (TRPL) measurements were conducted on FTO/c-TiO_2/m-TiO_2 substrates. Decay curves are presented in Figure S6. All the curves were fitted with a triple exponential function in order to obtain the best coefficient of determination R^2 possible. The function used and the fitting parameters extracted are gathered in Table S3. This allowed us to extract τ_{fast} and τ_{slow}, which represent, respectively, the non-radiative recombination at the ETL/PVK interface (reflecting the speed of charge injection in the transport layer) and the bimolecular recombination in the bulk of the perovskite (reflecting the bulk trap density). A higher τ_{slow} entails a much better bulk structural quality of the perovskite. In the meantime, a lower τ_{fast} value indicates better charge extraction at ETL/PVK layer, therefore, lower interface recombination centers and defect density. Two major features are observed. First, d-MAPI-HEA exhibits eight times higher τ_{slow} value than MAPI (148.63 ns vs. 15.62 ns respectively). This result emphasizes that d-MAPI-HEA perovskite has better bulk quality and homogeneity. The use of additives markedly increases the quality of the layer. It is in good agreement with the XRD results observed in Figure 2d. Second, d-MAPI-HEA also has a higher τ_{fast} value of 2.60 ns than MAPI, which presents a τ_{fast} value of 0.72 ns (≈ 4 times higher). This result reflects a lower quality of the junction TiO_2/PVK interface, resulting in a higher recombination center density at ETL/PVK interface for d-MAPI-HEA. This downgraded interface, rooting numerous recombination, thus leads to

poor charge extraction, which explains the lower J_{SC} and *FF* values of d-MAPI-HEA and the underestimation of the J_{SC} observed by EQE. It also suggests a slower electron injection that leads to a charge accumulation that can accentuate the hysteresis phenomenon.

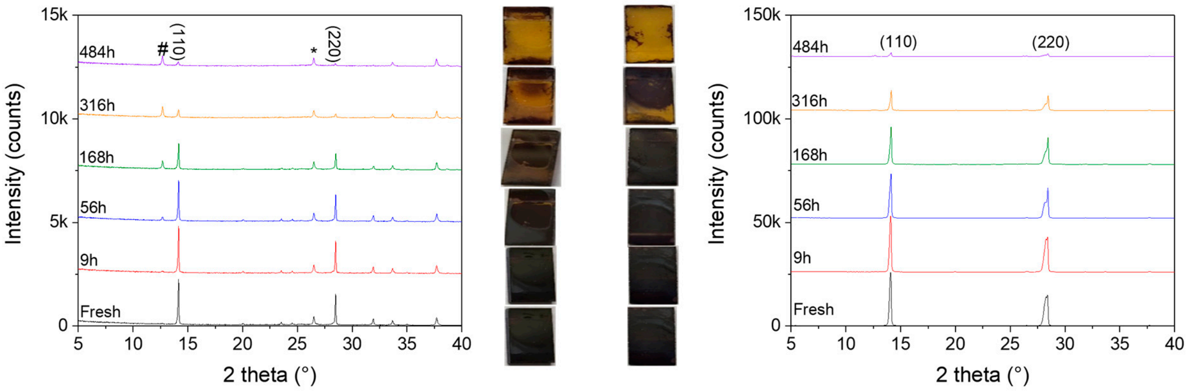

Figure S7. XRD patterns from the stability test (50–70 % RH, T°= 15–20 °C) of MAPI (left) and d-MAPI-HEA (right) films. FTO peaks are indicated by the * symbol. # indicates PbI_2. XRD corresponding film images upon their aging are displayed in the middle panel.

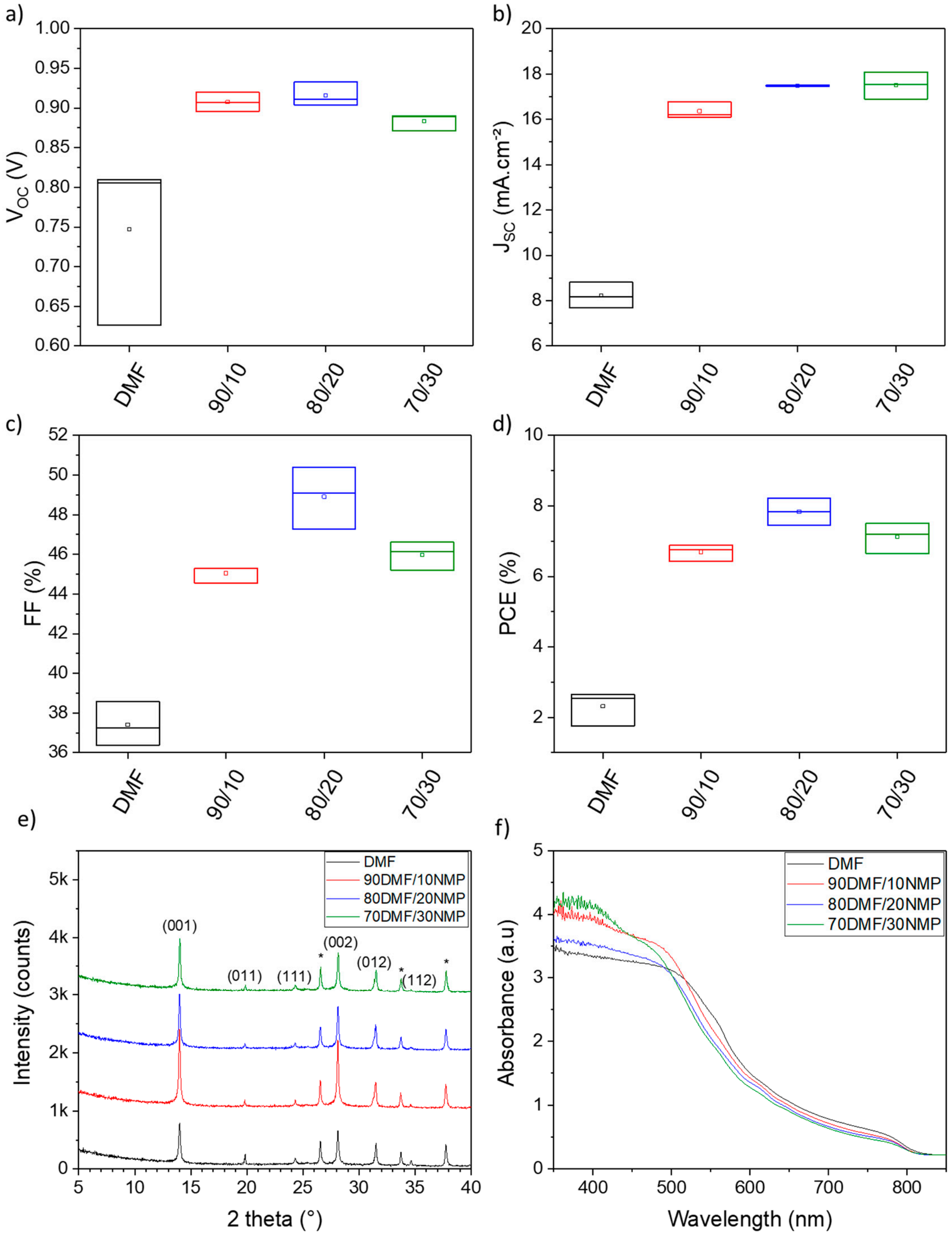

Figure S8. Box plots of d-FAPI-TEA devices for various DMF/NMP ratios (**a**) V_{OC}, (**b**) J_{SC}, (**c**) *FF*, and (**d**) *PCE* on the reverse scan. The x-axis indicates the volume ratio of DMF/NMP. Effect of DMF/NMP solvent volume ratios on: (**e**) XRD pattern (* indicates FTO) and (**f**) UV-Visible absorbance curves of d-FAPI-TEA perovskite films for various DMF/NMP ratios.

Table S4. *J-V* curves parameters, *PCE*s and *HI*s of d-FAPI-TEA PSCs with different DMF/NMP volume ratios.

Solvent	Scan direction	V_{OC} [V]	J_{SC} [mA.cm^{-2}]	*FF* [%]	*PCE* [%]	*HI* [%]
DMF	Reverse	0.81	8.82	37.25	2.68	64.2
	Forward	0.68	5.88	23.92	0.95	
90 DMF/10 NMP	Reverse	0.91	16.78	45.28	6.89	68.3
	Forward	0.75	16.21	17.98	2.19	
80 DMF/20 NMP	Reverse	0.93	17.49	50.37	8.22	61.1
	Forward	0.76	17.57	26.06	3.50	
70 DMF/30 NMP	Reverse	0.89	18.09	46.61	7.50	61.5
	Forward	0.73	18.18	25.18	3.36	

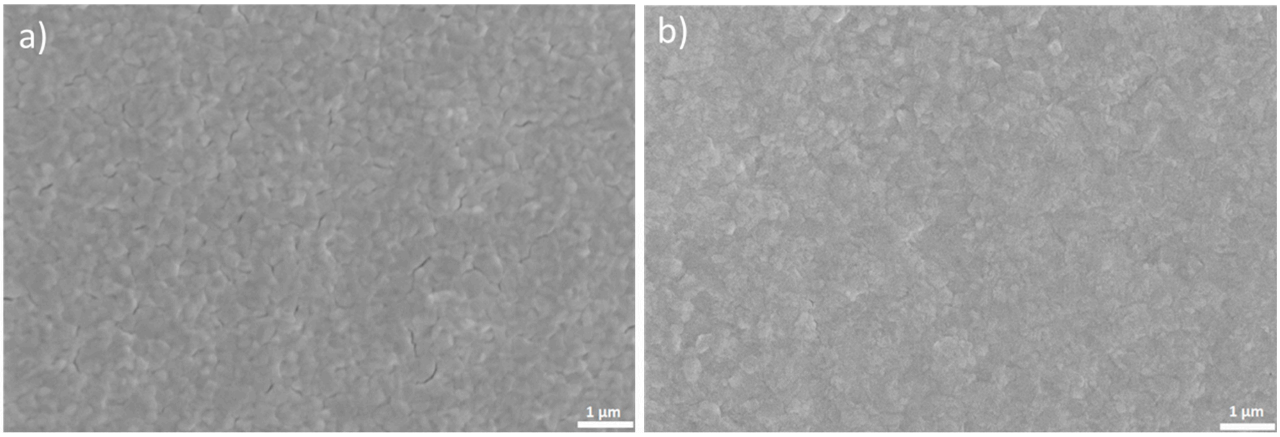

Figure S9. SEM images of d-FAPI-TEA layers prepared with (**a**) pure DMF solvent and (**b**) a mixture of 80DMF/20NMP solvents (vol%). Scale bar: 1 µm.

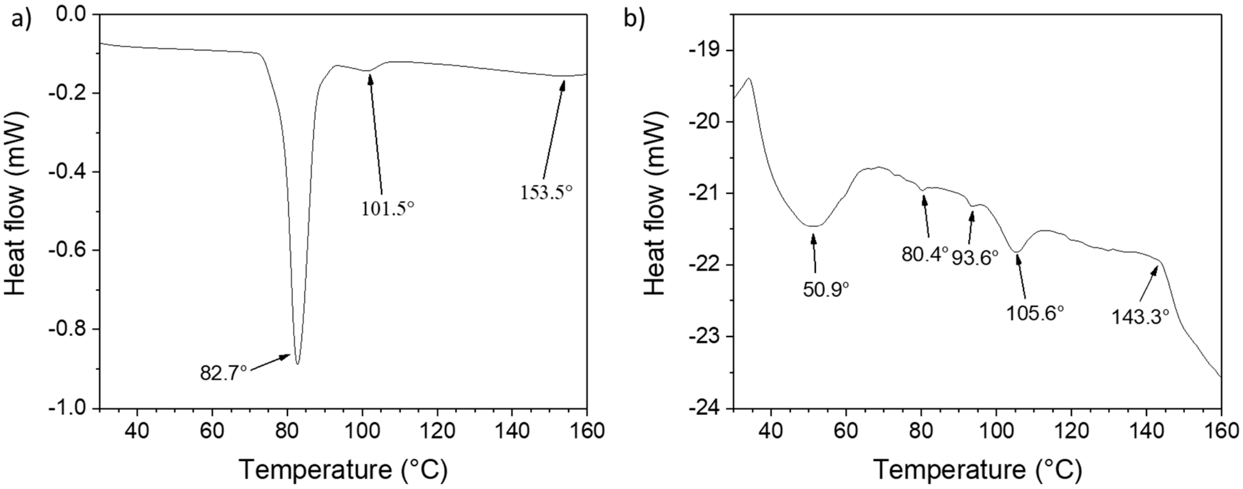

Figure S10. DSC curves of (**a**) FAPI and (**b**) d-FAPI-TEA.

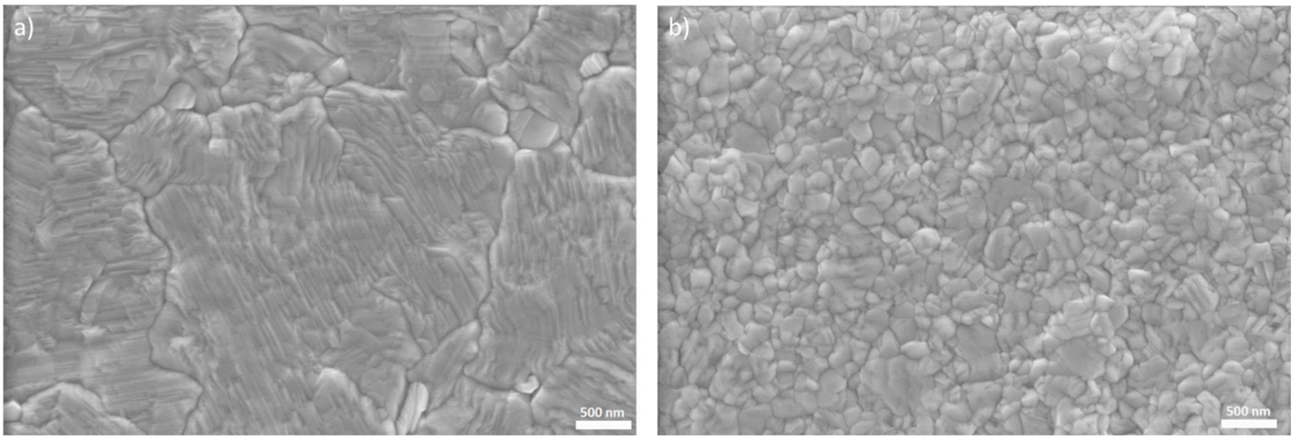

Figure S11. SEM images of (**a**) FAPI (**b**) d-FAPI-TEA. Scale bar: 500 nm.

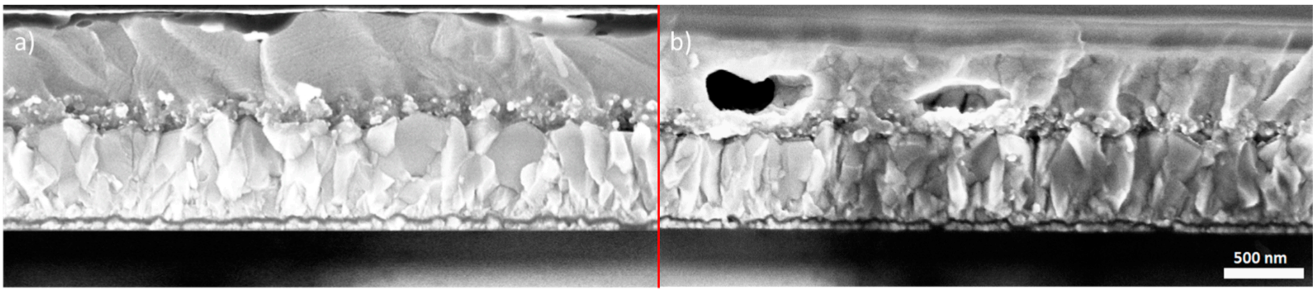

Figure S12. Cross-section SEM images of (**a**) FAPI and (**b**) d-FAPI-TEA. Au electrode was present on top of d-FAPI-TEA film. Scale bar: 500 μm.

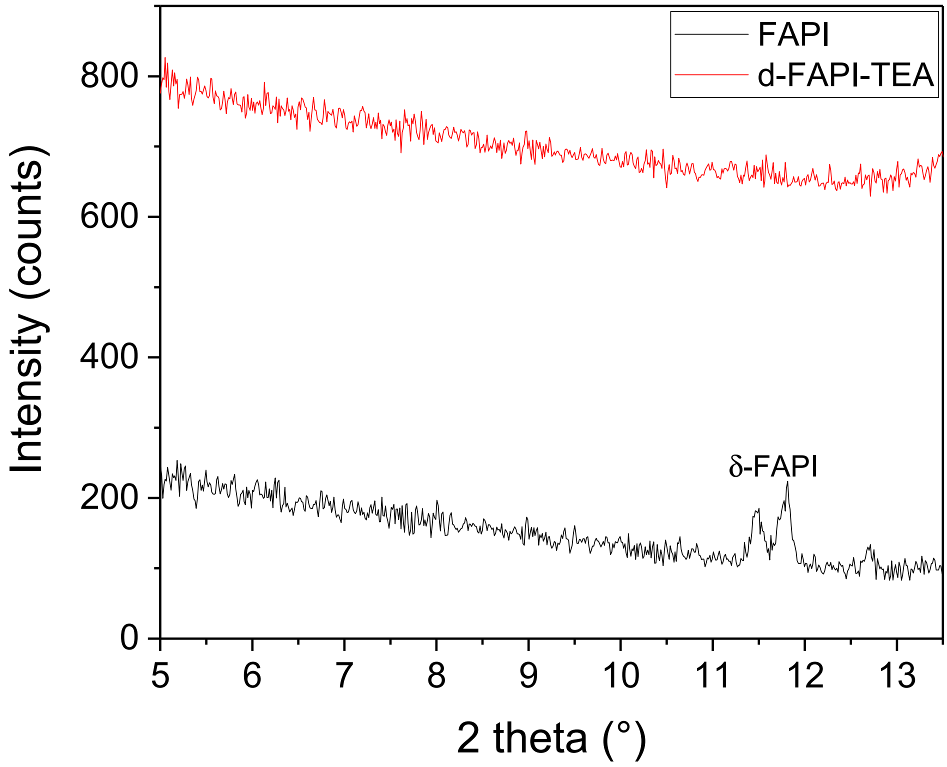

Figure S13. XRD patterns of FAPI and d-FAPI-TEA films zoomed between 5°–13.5°.

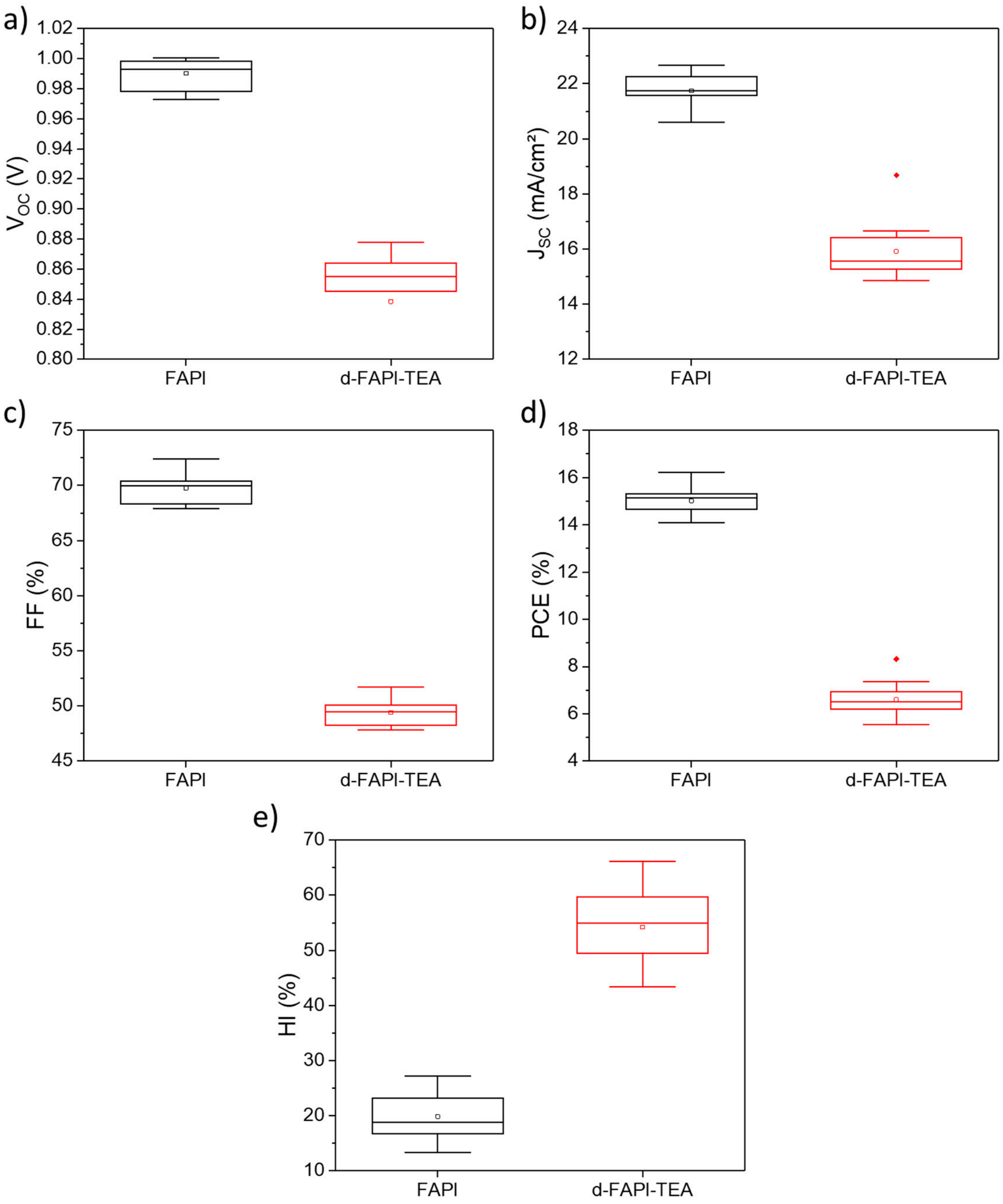

Figure S14. Box charts of FAPI and d-FAPI-TEA PSCs *J-V* curve parameters. (**a**) V_{OC}; (**b**) J_{SC}; (**c**) *FF*; (**d**) *PCE* measured on reverse scans; and (**e**) *HI*.

Table S5. *J-V* parameters, *PCE* and *HI* of best FAPI and optimized d-FAPI-TEA PSCs.

**Sample**	**Scan direction**	V_{OC} **[V]**	J_{SC} **[mA.cm^{-2}]**	***FF* [%]**	***PCE* [%]**	***HI* [%]**
FAPI	Reverse	0.99	22.63	72.40	16.23	17.9
	Forward	0.95	22.52	62.56	13.33	
d-FAPI-TEA	Reverse	0.88	18.68	50.79	8.33	43.4
	Forward	0.75	18.89	33.13	4.71	
